# Severity and duration of diabetic foot ulcer (DFU) before seeking care as predictors of healing time: A retrospective cohort study

**DOI:** 10.1371/journal.pone.0177176

**Published:** 2017-05-12

**Authors:** Hilde Smith-Strøm, Marjolein M. Iversen, Jannicke Igland, Truls Østbye, Marit Graue, Svein Skeie, Bei Wu, Berit Rokne

**Affiliations:** 1 Department of Health and Social Science, Centre for Evidence-Based Practice, Western Norway University of Applied Science, Bergen, Norway; 2 Department of Global Public Health and Primary Care, University of Bergen, Bergen, Norway; 3 Department of Medicine, Section of Endocrinology, Stavanger University Hospital, Stavanger, Norway; 4 Duke Global Health Institute, Duke University, North Carolina, Durham, United States of America; 5 Department of Research, Stavanger University Hospital, Stavanger, Norway; 6 Duke School of Nursing, Duke University, North Carolina, Durham, United States of America; 7 Rory Meyers College of Nursing, New York University New York, New York, United States of America; Centers for Disease Control and Prevention, UNITED STATES

## Abstract

**Objectives:**

To investigate whether A) duration of ulcer before start of treatment in specialist health care, and B) severity of ulcer according to University of Texas classification system (UT) at start of treatment (baseline), are independent predictors of healing time.

**Methods:**

This retrospective cohort study, based on electronic medical record data, included 105 patients from two outpatient clinics in Western Norway with a new diabetic foot ulcer during 2009–2011. The associations of duration of ulcer and ulcer severity with healing time were assessed using cumulative incidence curves and subdistribution hazard ratio estimated using competing risk regression with adjustment for potential confounders.

**Results:**

Of the 105 participants, 45.7% achieved ulcer healing, 36.2% underwent amputations, 9.5% died before ulcer healing and 8.5% were lost to follow-up. Patients who were referred to specialist health care by a general practitioner ≥ 52 days after ulcer onset had a 58% (SHR 0.42, CI 0.18–0.98) decreased healing rate compared to patients who were referred earlier, in the adjusted model. High severity (grade 2/3, stage C/D) according to the UT classification system was associated with a decreased healing rate compared to low severity (grade1, stage A/B or grade 2, stage A) with SHR (95% CI) equal to 0.14 (0.05–0.43) after adjustment for referral time and other potential confounders.

**Conclusion:**

Early detection and referral by both the patient and general practitioner are crucial for optimal foot ulcer healing. Ulcer grade and severity are also important predictors for healing time, and early screening to assess the severity and initiation of prompt treatment is important.

## Introduction

Diabetic foot ulcer is a feared complication of diabetes with a yearly incidence around 2–4% [1]. A diabetic foot ulcer has a variety of causes, often including peripheral ischemia, neuropathy or both. Ulcer healing takes weeks or months, and one-third of ulcers never heal with amputation as the consequence [2].

Factors affecting healing time include duration of ulcer, but limited research on the influence of duration of ulcer before treatment starts in specialist health care is available. Although some have investigated the associations between duration of ulcer before specialist health care treatment and healing time among persons with a diabetic foot ulcer [3–7], referral pathways are still not optimal. Many patients have delayed specialist health care referral due to lack of awareness of the potential consequences of a diabetic foot ulcer among patients and health care professionals and poor management strategies or ischemia detection [8]. In Norway, general practitioners coordinate medical follow-up and serve as “gate keepers” to specialist care, but still there are unclear referral practices between primary and specialist health care [9]. The importance of optimal referral patterns is also emphasised in international guidelines [10, 11]. However, to our knowledge, no studies have assessed the different periods of the referral pathway among individuals with diabetic foot ulcers. More evidence is therefore needed to assess the delay in referral pathways and the impact of these delays.

Diabetic foot ulcer treatment is challenging and time-consuming. Thus, predicting outcomes among patients with diabetic foot ulcers help clinicians to provide effective management strategies [12]. Using screening tools to identify vulnerable subgroups to detect diabetic foot ulcers at an early stage is important. However, the use of classification systems as a screening tool in clinical practice is scarce [13]. The University of Texas (UT) classification system is one of few systems that have been validated [13–15]. Although widely used, it is emphasised that more research is needed to assess to what degree this system reflects the population for which it is intended [16]. In Norway, a diabetic foot risk classification system has not yet been implemented in national guidelines. Thus, the UT classification system might be relevant for investigating predictors for healing time.

By utilizing a Norwegian cohort of foot ulcer patients from specialist health care outpatient clinics our main aim was to investigate the association of the following time fractions with healing time: the total duration of ulcer before start of treatment in specialist health care, defined as the time from patient-reported ulcer onset to start of treatment in specialist health care and two different fractions of duration of ulcer: 1) time from patient-reported ulcer onset to referral by general practitioner to specialist health care and 2) time from referral by general practitioner to start of treatment in specialist health care. In addition, we wanted to explore whether severity of the ulcer in terms of grade and stage at start of treatment in specialist health care was associated with healing time and whether duration of ulcer and severity showed independent associations after mutual adjustment and adjustment for other potential confounders.

## Material and methods

**Fig 1:**
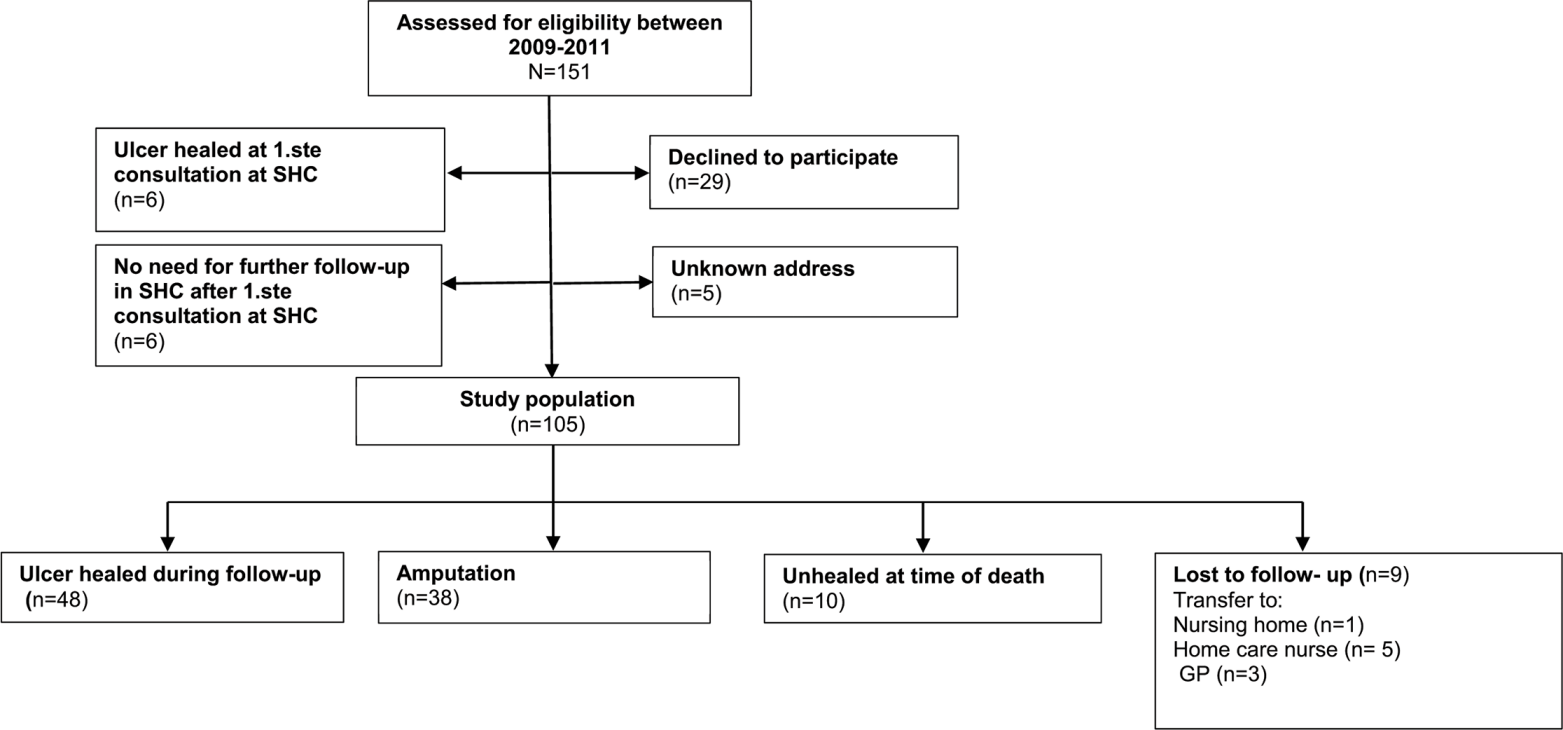
Flowchart: Study population.

This retrospective cohort study included all patients with a new diabetic foot ulcer presenting for the first time at two specialist outpatient clinics in Western Norway between 1 January 2009 and 31 December 2011 (Fig 1). In this study period, guidelines for foot assessment and treatment were provided through the National Professional Guideline for Diabetes—prevention, diagnosis and treatment (IS-1674) [17].

Patients previously treated for foot ulcers in specialist health care in the last 12 months before baseline were excluded. Each patient was followed to healing, amputation or death. A foot ulcer was defined as a skin lesion below the ankle. Participant information relating to baseline and follow up was obtained from medical electronic records. We recorded data on a standardised record form designed for this study and based on the research literature, clinical guidelines and expert opinions. A nurse specialized in diabetes and wound treatment from each outpatient clinic collected data from medical records. Data were collected between February 8, 2015 and January 2, 2016.

Missing values for the different variables are reported in Table 1. In the competing risk analysis missing data were addressed by listwise deletion. Overall, most of the information required was available, except for information on ulcer area. 41.9% of the patients did not have this information on ulcer area, thus, we decided not to include ulcer area as a study variable.

**Table 1:** Baseline characteristics of patients with a diabetic foot ulcer.

Characteristics	Total n = 105
***Demographic variables***	
Sex, n (%)	
Male	74 (70.5)
Age, years, mean, (SD)	68.7 (14.8)
***Disease-related variables***	
Diabetes type n (%)	
Type II	83 (79.0)
Insulin treatment, n (%)	
Did use insulin	68 (64.8)
HbA1c (mmol/l), Mean (±SD)	63 (±17.5)
HbA1c (%), Mean (±SD)	7.9 (±1.6)
Coronary diseases, n (%)	48 (45.7)
Neuropathy, n (%)	69 (65.7)
***Ulcer variables***	
Time from patient-reported ulcer onset to start of treatment in specialist health care, n (%)	
0–27 days	33 (31.4)
28–59 days	28 (26.7)
≥ 60 days	40 (38.1)
Missing	4 (3.8)
Time from patient-reported ulcer onset to referral by general practitioner to specialist health care, n (%)	
0–13 days	26 (24.8)
14–51 days	40 (38.1)
≥ 52 days	33 (31.4)
Missing	6 (5.7)
Time from referral by general practitioner to start of treatment in specialist health care, n (%)	
0 days	26 (24.8)
1–13 days	41 (39.1)
≥ 14 days	36 (34.3)
Missing	2 (1.9)
Localization of ulcer, n (%)	
Toe	64 (61.0)
Metatarsal/plantar	19 (18.1)
Heel	22 (21.0)
Vascular surgical treatment	
Percutaneous transluminal angiography /Bypass	26 (24.8)

### Main exposures

The main exposure variables were duration of ulcer and ulcer severity. Duration of ulcer was defined as the time from patient-reported ulcer onset to start of treatment in specialist healthcare using the tertiles: 0–27 days, 28–59 days and ≥ 60 days and further divided into two periods: 1) time from patient-reported ulcer onset to referral by general practitioner to specialist health care (divided into three groups using the tertiles: 0–13 days, 14–51 days and ≥ 52 days) and 2) time from referral by general practitioner to start of treatment in specialist health care (divided into three groups using the tertiles: 0 days, 1–13 days and ≥ 14 days). There are no established cut-off criteria for defining short, medium and long referral time; therefore, we chose to use tertiles to avoid biased cut-offs.

Ulcer severity was classified according to the UT classification system [15, 18] as grade 1 (superficial wound not involving tendon, capsule or bone), grade 2 (penetrating to the tendon or capsule) or grade 3 (penetrating to the bone or joint). Patients with ulcer grade 0 (completely healed ulcer) were excluded. Stages were: clean wounds (stage A); non-ischemia, infected (stage B); ischemia, non-infected (stage C); or ischemia, infected (stage D). Because of the small numbers in some categories, we combined grade and stage into three categories defined as low severity, medium severity and high severity determined from a clinical perspective. Low severity was defined as Grade 1 + stage A/B or grade 2 + stage A. Medium severity was defined as: Grade 1 + stage C/D or grade 2 + stage B or grade 3 + stage A/B and high severity was defined as grade 2/3 combined with stage C/D.

If the patient had multiple ulcers, the most severe ulcer (according to UT classification system), was selected as the index ulcer. This selection was made before collecting data on whether the ulcer healed.

### Demographic and clinical variables

Demographic and clinical variables which were considered to be potential confounders were sex, age, HbA1c, coronary disease, vascular surgical treatment, and neuropathy. These variables were selected based on previous literature and clinical judgement. Age was defined as the age at first consultation at the outpatient clinic. HbA1c measurements were reported in the International Federation of Clinical Chemistry units (mmol/mol) in addition to derived NGSP units (%) upon attendance at the outpatient clinic. Coronary disease was defined as having angina pectoris, history of myocardial infarction, previous coronary angioplasty or artery coronary bypass operation. Vascular surgical treatment includes information on percutaneous transluminal angiography of the peripheral arteries or bypass. Neuropathy was defined as an abnormal pressure sensation evaluated with the 10-g monofilament [19].

### Outcome, competing events and follow-up time

The outcome was healing time, defined as the time from the start of treatment in specialist health care until ulcer healing. Healing was defined as healing (intact skin) of the whole foot without any surgery in the period of study. Amputation and death were considered competing events. Follow-up time was calculated as time from the date of inclusion (= treatment start in specialist health care) until healing, amputation, death or loss to follow-up, whichever came first. Amputation performed below the ankle was defined as minor amputation, whereas amputation above the ankle was defined as major amputation [20].

### Statistical analysis

Descriptive statistics for the study population at baseline were calculated as mean, standard deviations, counts and percentages. Tests for associations between categories of referral time and categories of ulcer severity were conducted using chi-square tests. Cumulative incidence functions for healing time were calculated using the *stcompet* command in Stata, with amputation and death treated as competing events. Cumulative incidence functions were calculated separately for duration of ulcer divided into two periods, and for the three combinations of grade and stage. Fine & Gray competing risk regression analysis [21] were used to calculate the association of duration of ulcer, ulcer severity classified according to the UT classification system and healing time, and association between amputation and ulcer severity. Amputation and death were treated as competing events in the subdistribution hazard regression model while loss to follow up were treated as censored observations [22]. Results were reported as sub distribution hazard ratio (SHR) with 95% confidence intervals.

We investigated the associations of predictors, potential confounders and the outcome using univariate competing risk regression models (model 1). Then, we constructed a model where the main exposures, the two factions of duration of ulcer and ulcer severity, were mutually adjusted (model 2). Finally, we constructed a multivariate competing risk regression model including potential confounders, such as age, sex, HbA1c, coronary disease, vascular surgical treatment and neuropathy, in addition to the two fractions of ulcer duration and ulcer severity (model 3). Potential deviations from the proportional hazards assumption were investigated by including covariates as time-dependent covariates in the model. No significant time-dependent effects were found.

Statistical significance was defined as P < 0.05 in all analyses. SPSS version 22 was used for the description of baseline data, and Stata version 14 was used for competing risk regression and to construct cumulative incidence function curves in competing risk analyses.

### Ethics

The study was approved by the Western Norway Regional Committee for Medical and Health Research Ethics (2011/1609). Study information was sent to all participants still alive at registration, and informed consent was obtained.

## Results

### Subjects characteristics

In total, 151 participants with a diabetic foot ulcer were identified from 2009–2011, and 46 patients were excluded because they did not meet the inclusion criteria; ulcer healed at first consultations at the specialist health care clinic, (n = 6), no need for further follow-up in specialist health care at first consultation in specialist health care (n = 6), declining to participate (n = 29) and unknown address (n = 5). Thus, the study sample comprised 105 patients (Fig 1). The clinical characteristics of the patients are reported in Table 1. The average age among the patients was 68.7 years (SD ±14.8), 70.5% were men, 79% had type 2 diabetes with a mean HbA1c 7.9% (SD ±1.6). Coronary disease and neuropathy were present in 45.7% and 65.7% of patients respectively, and 38.1% had an ulcer duration of 60 days or more before the start of the treatment at the specialist outpatient clinic (Table 1).

The association between the three-category ulcer severity variable and time from patient-reported ulcer onset to referral by general practitioner to specialist health care is shown in Table 2. The association was significant (P = 0.042) with a higher proportion with short duration time from ulcer onset until referral among those with less severe ulcers (50%), compared to those with more severe ulcers (34.9%). Fifty percent of patients with low ulcer severity had ulcer duration of 0–13 days before referral to specialist health care, while only 16% of patients with high severity had 0–13 days duration before referral. In the group with high severity, 34.9% of the patients had waited 52 days or more before referral. Corresponding tests for the other two referral time variables showed no significant associations with ulcer severity.

**Table 2:** Association between severity of ulcer according to the UT classification system and time from patient-reported ulcer onset to referral by general practitioner to specialist health care.

Time from PRUO^1^ to referral by GP^2^ to SHC^3^	Low severity	Medium severity	High severity	Total	p
0–13 days	12 (50.0)	7(21.9)	7 (16.3)	26 (26.3)	0.042
14–51 days	6 (25.0)	13 (40.6)	21 (48.8)	40 (40.4)	
≥ 52 days	6 (25.0)	12 (37.5)	15 (34.9)	33 (33.3)	
Total	24 (100)	32 (100)	43 (100)	99(100)	

^1^PRUO = patient reported onset of ulcer

^2^GP = General practitioner

^3^SHC = Specialist health care

### Main exposures

Thirty-eight point one percent of patients had had an ulcer 60 days or more before the start of treatment in specialist health care, 31.4% of patients had had an ulcer for ≥ 52 days from patient-reported ulcer onset to referral by general practitioner to specialist health care, whereas 34.3% waited more than 14 days from referral by general practitioner to treatment start in specialist health care (Table 1).

Ulcer characteristics of patients with a diabetic foot ulcer according to UT classification system at baseline is presented in Table 3. Peripheral arterial disease, infection and ulcer penetrating to bone or joint were present in 24 (22.9%) patients (grade 3/stage D), of these, 20 underwent amputation (10 minor amputations, 10 major), 2 experienced complete ulcer healing and 2 died before the ulcer healed. No patients with grade 1, stage A underwent amputation (Table 3). The categorization of patients into low-medium and high severity is shown with shadings in the table.

**Table 3:** Ulcer characteristics of patients with a diabetic foot ulcer according to UT classification system at baseline.

Stage		Grade 1	Grade 2	Grade 3	Total
A	*Clean wound*	16 (15.2)	1(1.0)	1 (1.0)	18 (17.1)
B	PAD -, infection +	8 (7.6)	10 (9.5)	11 (10.5)	29 (27.6)
C	PAD +, infection -	8 (7.6)	5 (4.8)	6 (5.7)	19 (18.1)
D	PAD +, infection +	5 (4.8)	10 (9.5)	24 (22.9)	39 (37.1)
Total, n, (%)		37 (35.2)	26 (24.8)	42 (40.0)	105 (100)

PAD: Peripheral arterial disease.

Grade 0: Pre-or post-ulcerative lesion, Grade 1: Superficial wound, not involving tendon, capsule or bone, Grade 2: Wound penetrating to tendon or capsule, Grade 3: Wound penetrating to bone or joint.

White area: low severity, Light Grey area: medium severity, Dark grey area: high severity.

### Outcome

In total, 48 (45.7%) patients’ ulcers healed completely without preceding amputation (either major or minor) and 38 (36.2%) underwent amputation (24 minor amputations and 14 major amputation). Ten (9.5%) patients died before the ulcer healed and nine (8.5%) patients were lost to follow-up. The median follow-up time measured from start of treatment in specialist health care to end of follow-up was 67 days (SD ± 185.4) for the total sample (including those who healed, amputated, died and lost to follow up). Mean follow-up time was 130 days. The median time measured from start of treatment in specialist health care to ulcer healing, including only those who healed, was 75.5 days (SD 123.4). Mean healing time was 113 days.

#### Cumulative incidence curve

**Fig 2:**
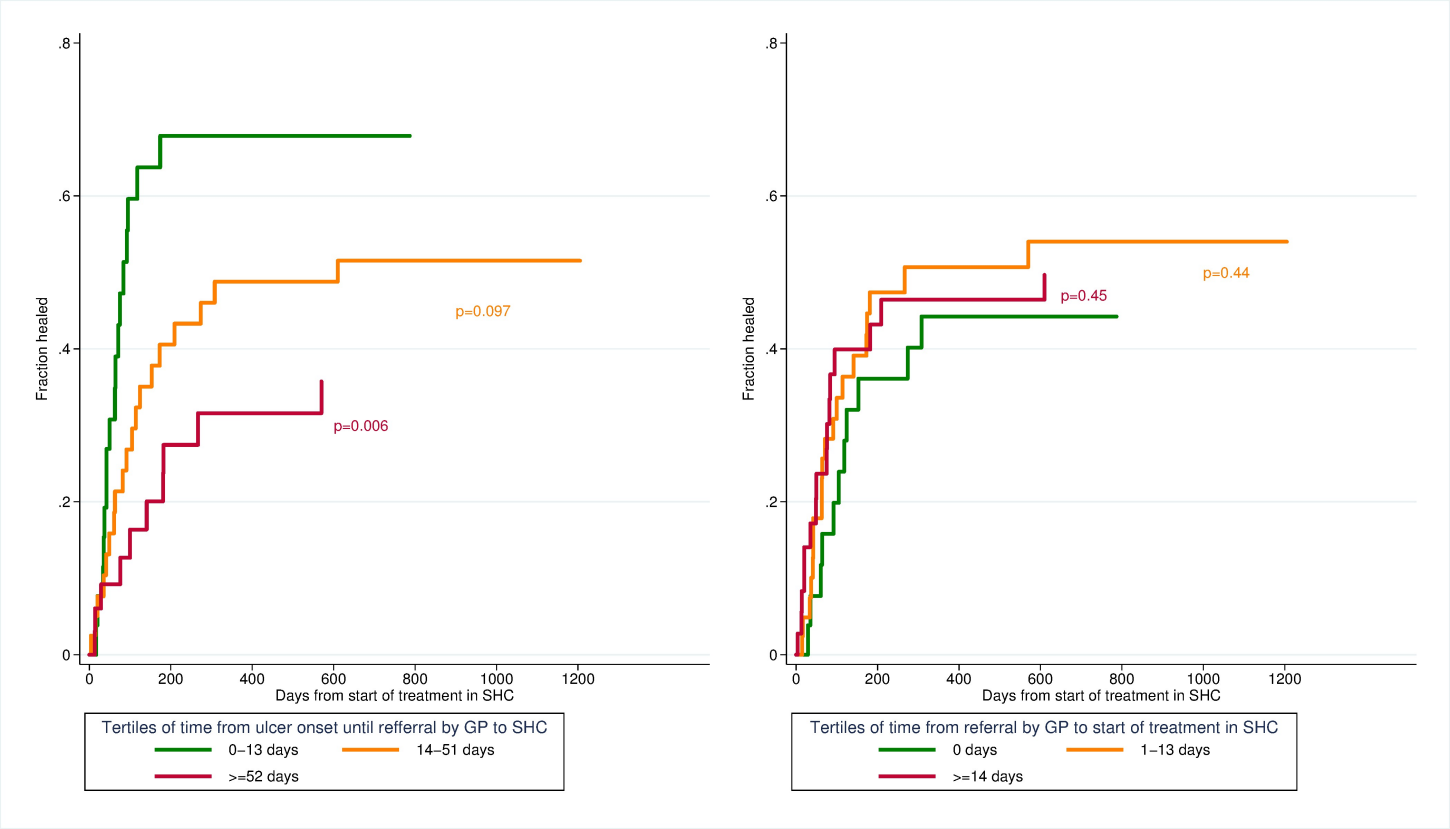
Cumulative incidence curves of healing time stratified by duration of ulcer. P-values from univariate competing risk regression.

Cumulative incidence curves of healing time stratified by duration of ulcer are shown in Fig 2. Patients in the upper tertile of time from ulcer onset to referral by general practitioner to specialist health care (≥ 52 days after ulcer onset) had increased healing time compared to earlier referral. There was no significant difference between the tertiles of time from referral by general practitioners to start of treatment in specialist health care.

**Fig 3:**
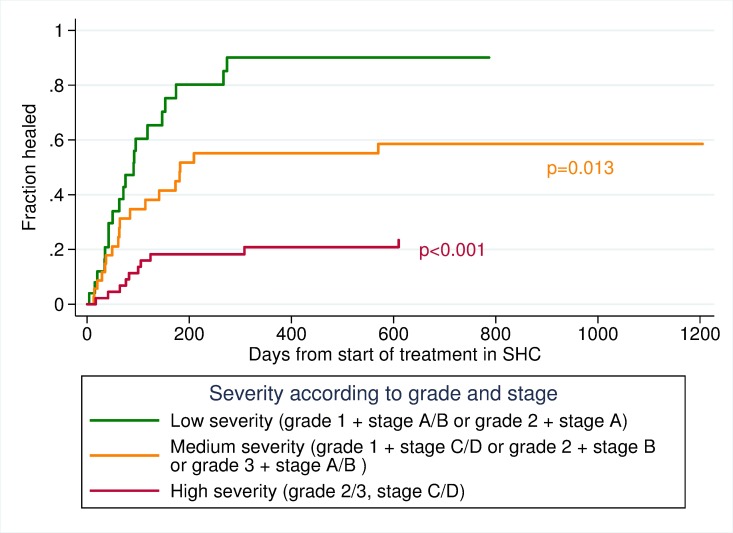
Cumulative incidence curves of healing time stratified by severity of ulcer. P-values from univariate competing risk regression.

Cumulative incidence curves of healing time stratified by severity of ulcer (levels of grade and stage), are seen in Fig 3, which shows an increased healing time for patients with a high severity of ulcer compared to the two other categories of grades and stages.

#### Univariate competing risk regression analysis

The total duration of the ulcer from ulcer onset to start of treatment in specialist health care showed no significant association with healing time (SHR 0.62, CI 0.30–1.28). When duration of ulcer was divided into two periods, there was no association with time from general practitioners’ referral to specialist health care to start of treatment and healing time, but there was a strong association between the time from patient-reported ulcer onset to referral by general practitioner to specialist health care with healing time. Patients who were referred to specialist health care by a general practitioner 52 days or more after the onset of ulcer had a 67% (SHR 0.33, CI 0.15–0.72) decreased rate of healing compared to those referred earlier. Older age and vascular surgical treatment were also associated with a decreased rate of healing time (Table 4, model 1).

**Table 4:** Subdistribution hazard regression model to calculate the association between duration of ulcer, severity of ulcer and healing time.

	Total (n = 105) ulcer healed (n = 48)	Model 1 SHR (95% CI) Unadjusted	Model 2 SHR (95% CI) Mutually adjusted	Model 3 SHR (95% CI) Full model
Time from patient-reported ulcer onset to start of treatment in specialist health care				
0–27 days	33/16	1		
28–59 days	28/18	1.58 (0.81–3.08)		
≥ 60	40/13	0.62 (0.30–1.28)		
Time from patient-reported ulcer onset to referral by general practitioner to specialist health care				
0–13 days	26/17	1	1	1
14–51 days	40/19	0.57 (0.29–1.11)	1.00 (0.52–1.93)	1.16 (0.51–2.62)
≥ 52 days	33/10	0.33 (0.15–0.72)	0.38 (0.17–0.86)	0.42 (0.18–0.98)
Time from referral by general practitioner to start of treatment in specialist health care				
0 days	26/11	1	1	1
1–13 days	41/20	1.33 (0.66–2.67)	1.45 (0.68–3.09)	1.56 (0.62–3.90)
≥ 14 days	36/16	1.30 (0.61–2.76)	1.76 (0.83–3.77)	1.84 (0.70–4.84)
Severity of ulcer classified after UT classification system				
Low severity	20/25	1	1	1
Medium severity	18/35	0.45 (0.24–0.85)	0.45 (0.23–0.91)	0.45 (0.23–0.88)
High severity	10/45	0.13 (0.06–0.28)	0.14 (0.06–0.30)	0.14 (0.05–0.43)
Age	105/48	0.98 (0.96–1.00)		1.00 (0.98–1.03)
Sex				
Male	74/35	1		1
Female	31/13	0.85 (0.46–1.58)		0.66 (0.36–1.23)
HbA1c	105/48	0.98 (0.82–1.18)		1.12 (0.87–1.44)
Coronary disease				
No	57/26	1		1
Yes	48/22	0.85 (0.49–1.48)		1.04 (0.55–1.96)
Vascular surgery treatment				
Yes	26/5	0.24 (0.10–0.59)		0.59 (0.19–1.79)
No	79/43	1		1
Neuropathy				
Yes	69/35	1.33 (0.70–2.51)		1.05 (0.53–2.07)
No	36/13	1		1

High ulcer severity ulcer had 87% (SHR 0.13, CI 0.06–0.28) decreased rate of healing compared to low severity. Ulcer of medium severity had 55% decreased rate of healing compared to ulcer with low severity (SHR 0.45, CI 0.24–0.85) (Table 4, model 1). Competing risk analyses with amputation as the endpoint showed a significant association for ulcer severity with three times higher risk of amputation in the category with high severity compared to the category with low severity (SHR 3.15, CI 1.49–6.66) (results not shown in tables). We did not observe any significant associations between total duration of ulcer and risk of amputation or between time from patient-reported ulcer onset to referral by general practitioner to specialist health care and risk of amputation (results not shown in tables). We did however observe a significant association between time from general practitioners’ referral to specialist health care to start of treatment and risk of amputation with a lower risk of amputation among those who waited more than 14 days compared to those who had their first appointment the same day as they were referred (SHR 0.41, CI 0.18–0.94). Among the 26 patients having their first appointment the same day as they were referred, 54% ended up with a minor or major amputation.

#### Multivariate analysis

Estimated SHRs increased slightly for patients who had an ulcer ≥ 52 days from 0.33 to 0.38 when the following variables were included in the same model: time from patient-reported ulcer onset to referral by general practitioner to specialist health care, time from referral from general practitioner referral to start treatment in specialist health care and ulcer severity (Table 4, model 2). When age, sex, HbA1c, coronary disease, vascular surgical treatment and neuropathy were entered into the model, the SHRs for time from patient-reported ulcer onset to referral by general practitioner to specialist health care, time from referral from general practitioner to start of treatment in specialist health care and ulcer severity did not change markedly. Age and vascular surgical treatment were associated with reduced healing time in the univariate analysis, but the association did not remain significant after adjustment in the multivariate analysis (Table 4, model 3). For ulcer severity, the association was still significant after adjustment for both duration of ulcer and potential confounders. The significant association between time from referral from general practitioner to start of treatment in specialist health care and risk of amputation observed in the univariate competing risk model was no longer significant after adjustment for severity of the ulcer.

## Discussion

Time from patient-reported ulcer onset to referral by general practitioner to specialist health care and the two highest levels of ulcer severity were independently associated with healing time for diabetic foot ulcer while controlling for age, sex, HbA1c, coronary disease, vascular surgery treatment and neuropathy.

The results show that duration of ulcer before starting specialist health care treatment influenced healing time, with time from onset of ulcer to referral by the general practitioner as the main contributor to the association. The waiting time between referral and start of treatment in specialist health care did not show a significant association with healing time and SHR’s were actually greater than 1. reflecting a tendency to higher probability of healing among those who waited ≥14 days for an appointment. This could possibly partly be explained by the observed inverse association between waiting time and risk of amputation, with significantly higher risk of amputation among those who waited 0 days compared to those who waited ≥14 days, leaving fewer patients behind to experience healing. Among the 26 patients who had their first appointment in specialist health care the same day as the referral, 61.5% had a wound in stage C or D and 54% ended in amputation, indicating very severe ulcers. The lack of an association between total duration of ulcer and healing time could possibly also be explained by the tendency to an association in the opposite direction for the second part of the duration time.

Margolis and colleagues (2002) [3] evaluated the association between different risk factors and healing time among 31,106 participants with neuropathic foot ulcers. They found that increased wound duration before initial treatment start in specialist health care was one of the major factors associated with reduced healing. However, these results were not supported in a UK study of 449 participants with diabetic foot ulcers [4]. These authors speculated whether this was caused by the fact that the date of ulcer onset simply was recorded by month. In the Eurodiale study, the variation between countries was considerable, with inconclusive results concerning healing time [2, 23]. In a recent British report (2016), findings indicated that the ulcer healing time increased compared to shorter interval if the interval to first assessment by specialist was > 2 months after ulcer onset [7]. Although the number of patients in our study was relatively small, we found that longer duration of ulcer before specialist health care treatment was associated with decreased healing rate. Our results underscore that the interval from patient-reported ulcer onset to specialist health care referral by a general practitioner seems more important than the interval between referral and the start of specialist health care treatment. In the Norwegian health care system, general practitioners are responsible for coordinating medical follow-up [24]. However, we did not find that more superficial DFU took longer for referral, but rather the opposite. Fifty percent of patients with low severity of the ulcer had an ulcer duration of 0–13 days before referral to SHC while only 16% of patients with high severity had 0–13 days duration before referral. In the group with high severity, 34.9% of the patients waited 52 days or more before referral. It is difficult to explain the reasons for this finding. It might be that patients with more severe ulcers waited for a long time before contacting the GP or that the GP tried to treat the ulcer before referring the patient to the specialist health care. We lack information of both these aspects. However, the data give valuable information of the importance of early referral to specialist health care to avoid severe complications. Therefore, it is important to communicate to patients and health care professionals in primary health care that referral pathways and adequate access to general practitioner services are crucial. Reduced function and further adverse complications can be prevented if ulcers are identified at an early stage [8, 10, 25]. A better follow-up strategy in primary health care and models that facilitate communication across different care levels should be considered.

Delayed specialist health care treatment start was seen in many patients, although guidelines stress the importance of early treatment to avoid adverse complications [10, 11]. In our cohort, 38.1% of patients had a duration of ulcer ≥ 60 days (2 months) prior to the start of specialist health care treatment. This is comparable with the results of the Eurodiale study involving 14 countries, where over 27% of participants were treated for >3 months before initial specialist health care treatment [8], while only 7.7% among patients with DFU in England and Wales had more than 60 days (2 months) delayed referral time to specialist health services [7]. Although substantial differences among countries exist, current guidelines were not followed when treating a significant number of patients [8, 11]. Our study showed a strong association between delayed referral to a specialist unit and healing time after adjustment for potential confounder with clear implications for routine care. Treatment is effective, guidelines are available and early intervention seems to reduce the burden of an adverse outcome.

The present cohort has a higher incidence of amputation and relative low incidence of ulcer healing compared to other studies [6, 8, 23]. In total, 52% of the ulcers leading to amputation were affected by infection, peripheral artery disease and ulcers penetrating to bone and joint These more severe risk factors may have had an impact on the relative high incidence of amputation. One other possible explanation might be that our definition of healing did not include minor amputation, which is in contrast to some other studies [6, 26]. In these studies, minor amputation could be regarded as a strategy leading to healing.

Ulcers of the highest and medium stage and grade were strongly associated with decreased rate of healing. Both peripheral arterial disease, independently and in combination with infection, are known predictors of ulcer healing leading to prolonged healing time. Patients with the combination of PAD, infection and ulcer penetrating to bone or joint were also more likely to undergo amputation than those with less severe ulcer stage [2, 23, 27]. In our cohort, PAD, infection and ulcer penetrating to bone or joint with amputation as an endpoint were seen in 20 of the patients. Given the association between severity of ulcer and healing time, early screening of people with a new ulcer is imperative to assess the severity and initiate adequate treatment to reduce the risk of amputation [2, 27].

We found an association between severity of ulcer and duration of ulcer, but the duration of ulcer and severity of ulcer still showed significant associations with healing time after mutual adjustment and adjustment for potential confounders. First, the persistent associations after adjustment for duration of ulcer indicate that ulcer severity at the first specialist health care consultation was important for healing time, regardless of how long the ulcer had lasted before the first consultation. In other words, an ulcer with a severe grade and severe stage has an increased healing time, even if it did not last long before start of treatment. Second, the independent association for duration of ulcer indicates that duration of ulcer affects healing through mechanisms other than greater ulcer severity. Other possible factors might be the quality of general practitioners’ treatment and a lack of health awareness among this patient group.

There are several limitations in this retrospective cohort study. First, the sample size is relatively small, which limit the statistical power. However, these results may still provide new knowledge about independent predictors for healing time and implications for further research. Second, in total 69.5% of the potential participants were included in the study. Non-participants might have been in worse health status, and this could potentially lead to selection bias. The increased healing time associated with duration of ulcer before start of treatment in specialist health care and severity of ulcer in the present study might therefore have been underestimated. Third, we acknowledge that the UT classification system omits reference to ulcer area. In our study we were not able to examine the impact of ulcer areas on healing due to missing data on ulcer size (cm^2^) (41.9%). In the time period that data were collected, it was not common to use pictures to measure ulcer area, which may explain the high occurrence of missing. As the UT classification system provides a standard description of an ulcer and help predict outcomes we decided only to use the UT classification system in the analyses. Fourth, the possible impact of early amputation as a strategy to obtain healing would be interesting to investigate, but this was not possible since follow-up was terminated at the time of minor or major amputation. Therefore, we do not know the healing time for patients who experienced ulcer healing after a minor amputation. Fifth, the incidence of amputation was high in this study, especially among those who had their first appointment in specialist health care the same day as they were referred by the GP, causing a non-significant increased rate of healing among those who waited longer for an appointment after referral was sent by the GP. In a population with a lower incidence of amputation, it might be more likely to observe an increased rate of healing with shorter waiting time, but the strength of the association would be weakened if patients with more severe ulcers have shorter waiting time. Sixth, information on how long the patient waited before he/she contacted a general practitioner was unavailable for most patients and could therefore not be included in the analysis. Finally, data on whether the general practitioner had treated the ulcer before the patient was referred to specialist health care was also lacking. Such information could provide important information on the causes of delayed referral, and further studies are necessary to assess the importance of these factors.

In summary, duration of diabetic foot ulcer before the start of treatment in specialist health care and ulcer severity influenced healing time independently of each other. Early identification of the ulcer by the patient and the general practitioner, as well as early referral by a general practitioner to specialist health care are important for ulcer healing and have clear implications for routine care. Grade and stage severity are important predictors for healing time. Early screening might identify patients needing extra support in treatment and follow-up care.

## Supporting information

S1 File Data set in excel format. (XLS)

S2 File Strobe checklist. (DOCX)

## Abbreviations

UT classification system: University of Texas classification system
PAD: peripheral arterial disease
SHR: subdistribution hazard ratio
GP: General practitioner
SHC: specialist health care
